# Intraoperative Extrication of a Pediatric Hand Entrapped Within a Household Meat Grinder: A Case Report

**DOI:** 10.7759/cureus.114658

**Published:** 2026-08-17

**Authors:** Katie Ross, Claire Carruthers, Martin Leblanc

**Affiliations:** 1 Division of Plastic and Reconstructive Surgery, Dalhousie University, Halifax, CAN

**Keywords:** case report, extrication, hand amputation, meat grinder injury, pediatric trauma

## Abstract

Hand injuries involving meat grinders are uncommon and frequently result in severe crush injury requiring amputation or complex reconstruction. This report describes a unique case of intraoperative mechanical extrication of a pediatric hand entrapped within a household meat grinder.

A right-hand-dominant six-year-old boy presented with his right hand entrapped in a household meat grinder. Due to mechanical entrapment and the lack of appropriate tools in the operating room, safe removal required intraoperative coordination with emergency services. The injury was non-reconstructable, requiring carpometacarpal-level amputation and multidisciplinary postoperative care.

This case illustrates the surgical and organizational challenges associated with pediatric meat grinder injuries and demonstrates the importance of rapid multidisciplinary collaboration. Prompt recognition of non-salvageable injury, decisive surgical management, and coordinated postoperative rehabilitation are critical to optimizing patient outcomes while reinforcing the importance of injury prevention.

## Introduction

Meat grinder-related hand injuries represent a rare but devastating subset of upper extremity trauma. Although uncommon in high-income countries, these injuries continue to occur in both domestic and occupational settings and are characterized by severe crush, avulsion, and rotational forces that frequently result in complex soft tissue destruction, contamination, and irreversible neurovascular injury [1-4]. Consequently, patients often require amputation or extensive reconstructive procedures despite advances in microsurgical techniques [3,5,6].

Pediatric patients are particularly susceptible to devastating injury because of their smaller hand size, which allows deeper engagement within the grinder mechanism [1,3]. Beyond the immediate surgical consequences, these injuries carry significant long-term functional, psychological, and developmental implications [7,8]. Previous reports have largely focused on injury patterns, reconstructive outcomes, and methods of emergency department or pre-hospital extrication, with relatively little emphasis on intraoperative management once conventional extraction techniques have failed [1,3,7-9].

Safe removal of an entrapped extremity presents unique technical challenges. Attempts at forceful extraction may worsen tissue destruction, while dismantling or cutting the device carries risks to both the patient and operating room personnel [5]. Successful management therefore requires balancing timely surgical intervention with preservation of remaining viable tissues and careful multidisciplinary coordination.

We present a rare pediatric case of complete hand entrapment within a household meat grinder requiring intraoperative mechanical extrication with assistance from municipal emergency services before definitive amputation. This report highlights not only the surgical management of a non-reconstructable injury but also the systems-level challenges encountered when specialized equipment unavailable within the operating room became necessary.

## Case presentation

A previously healthy six-year-old, right-hand-dominant boy presented as a trauma activation after his right hand became entrapped in a household meat grinder while assisting his father with food preparation. He was transported to the hospital with the hand still lodged within the device. On arrival, he was hemodynamically stable with no evidence of additional injuries on primary or secondary survey. Bleeding remained minimal while the hand was mechanically entrapped within the grinder, likely due to the device providing substantial pressure on the injured tissues.

**Figure 1:**
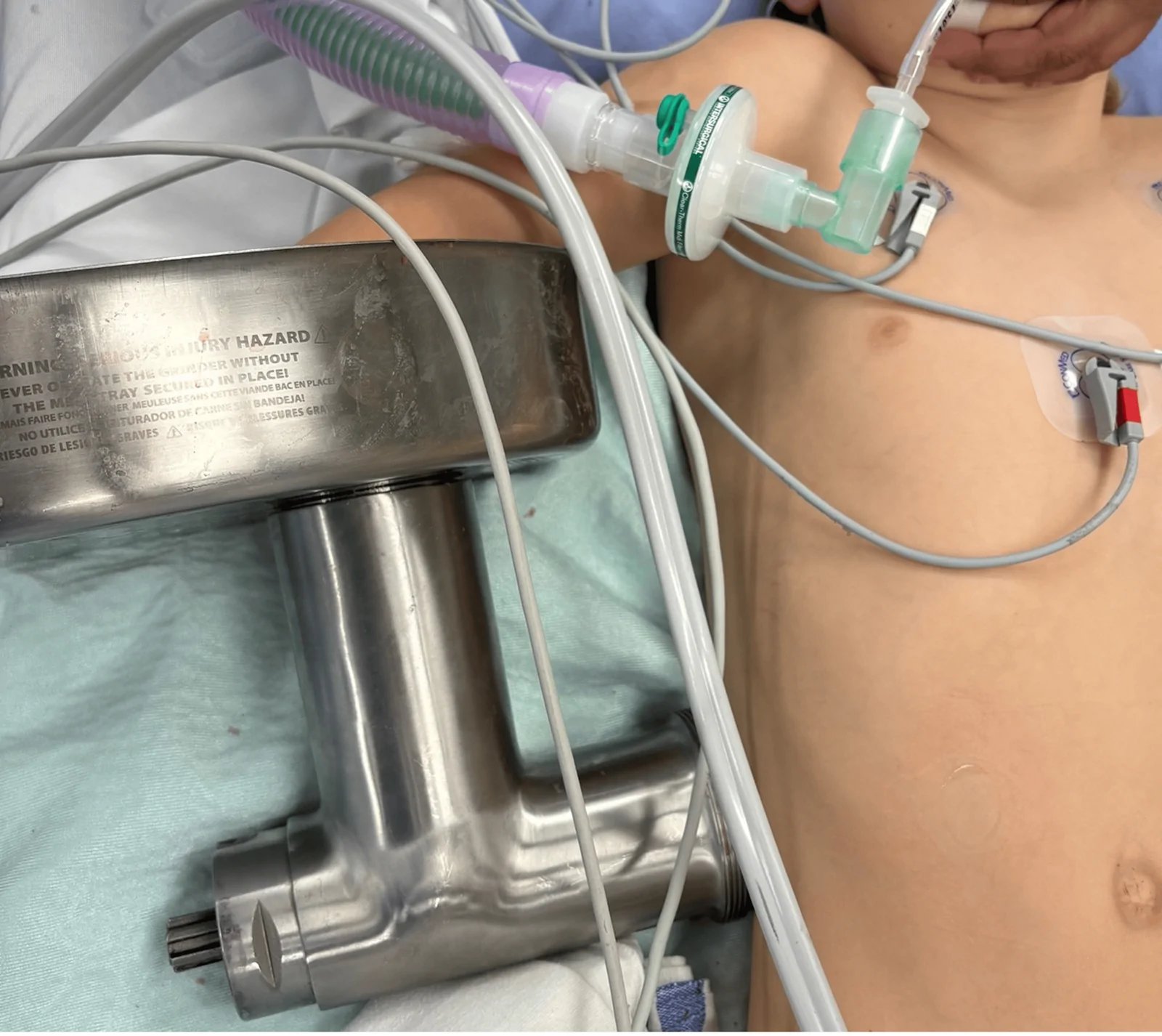
A 6-year-old boy with his right hand entrapped in a household meat grinder.

Given the mechanical entrapment and inability to safely extricate the limb in the emergency department, the patient was taken emergently to the operating room (Figure 1).

**Figure 2:**
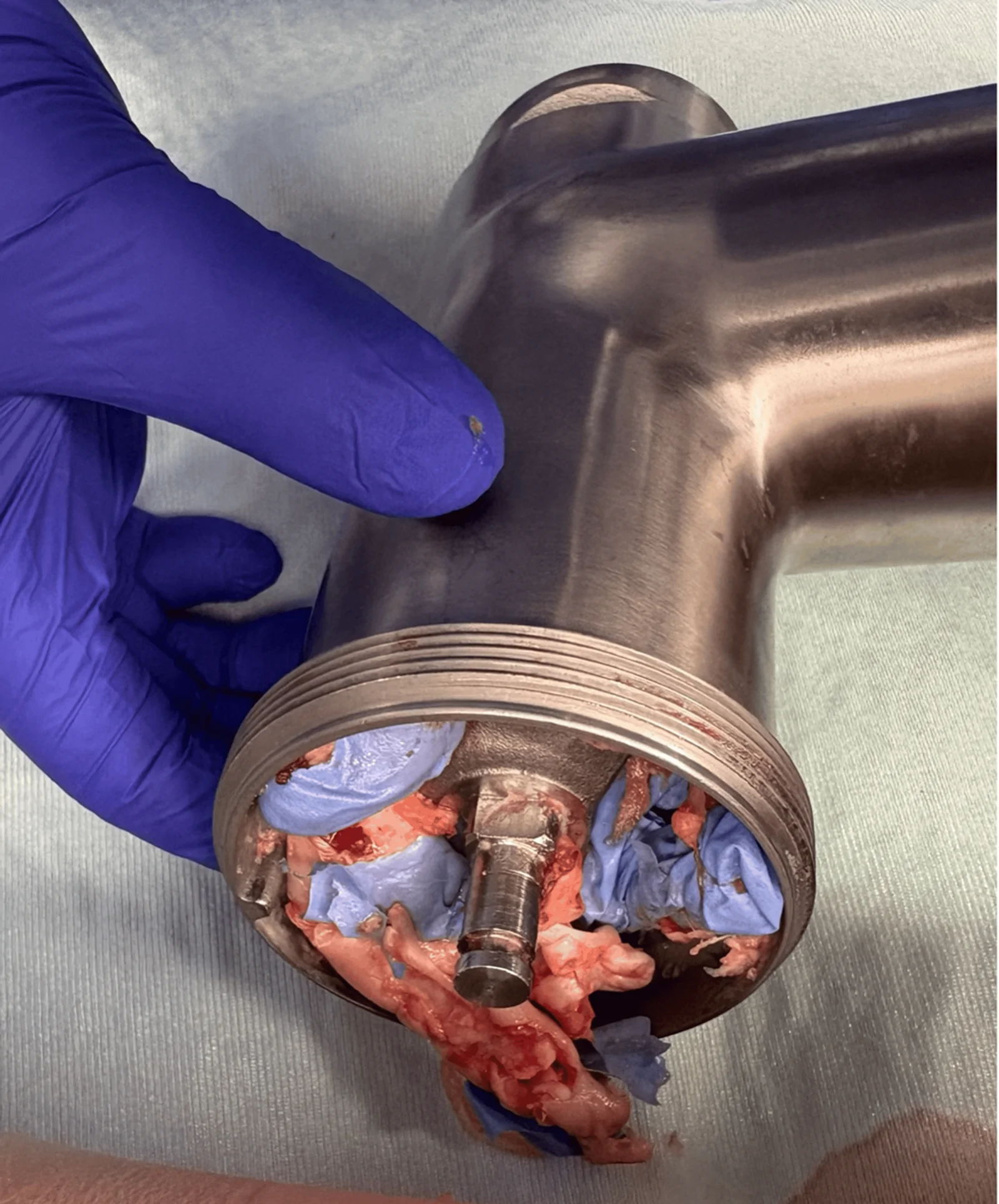
Mangled portion of the hand protruding from the distal end of the grinder, with extent of internal injury unclear.

After induction of general anesthesia and administration of intravenous antibiotics, examination of the device revealed a mangled portion of the hand protruding from the distal end of the grinder, with the extent of internal injury unclear (Figure 2).

Initial attempts at manual extraction, including gentle traction and attempted reversal of the grinder mechanism, were unsuccessful. Given the inability to free the limb and concern for further tissue injury, alternative strategies were considered. Due to the lack of appropriate cutting tools within the operating room, emergency services were contacted intraoperatively for assistance via 911. The patient was temporarily transferred-while intubated and monitored-to an adjacent procedure area to allow safe assessment by the fire department and access for equipment. Extraction options considered included using significant traction to free the hand and welding then prying the grinder open. Ultimately, the decision was made to use a large wrench and, with the assistance of the fire department, rotate the auger mechanism forward, allowing expulsion of the entrapped hand and completion of extraction. There was bleeding from the proximal stump, but limited oozing from the distal soft tissue, concerning for lack of viability of the tissue.

**Figure 3:**
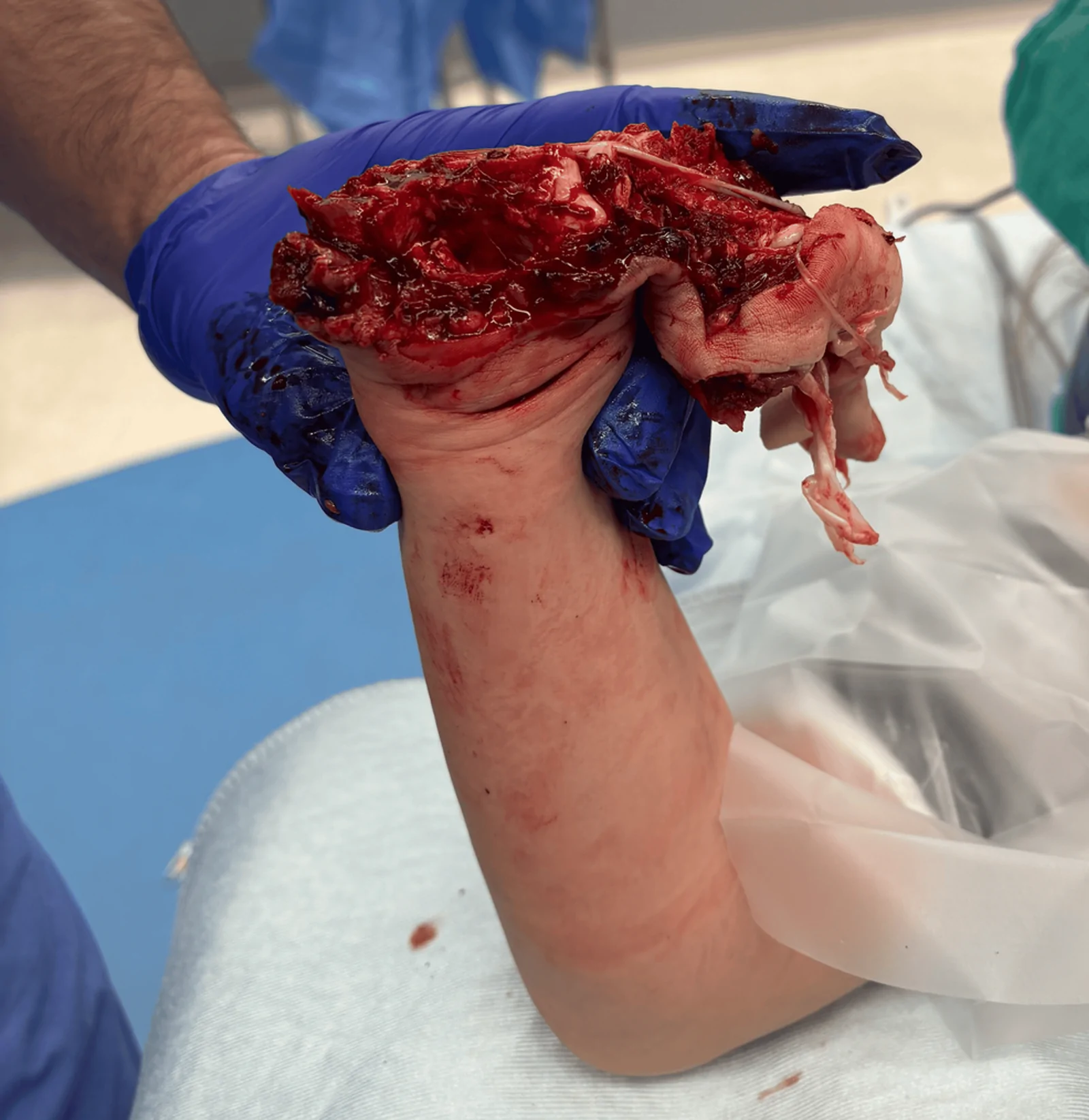
Non-viable soft tissue and comminuted bony injury.

**Figure 4:**
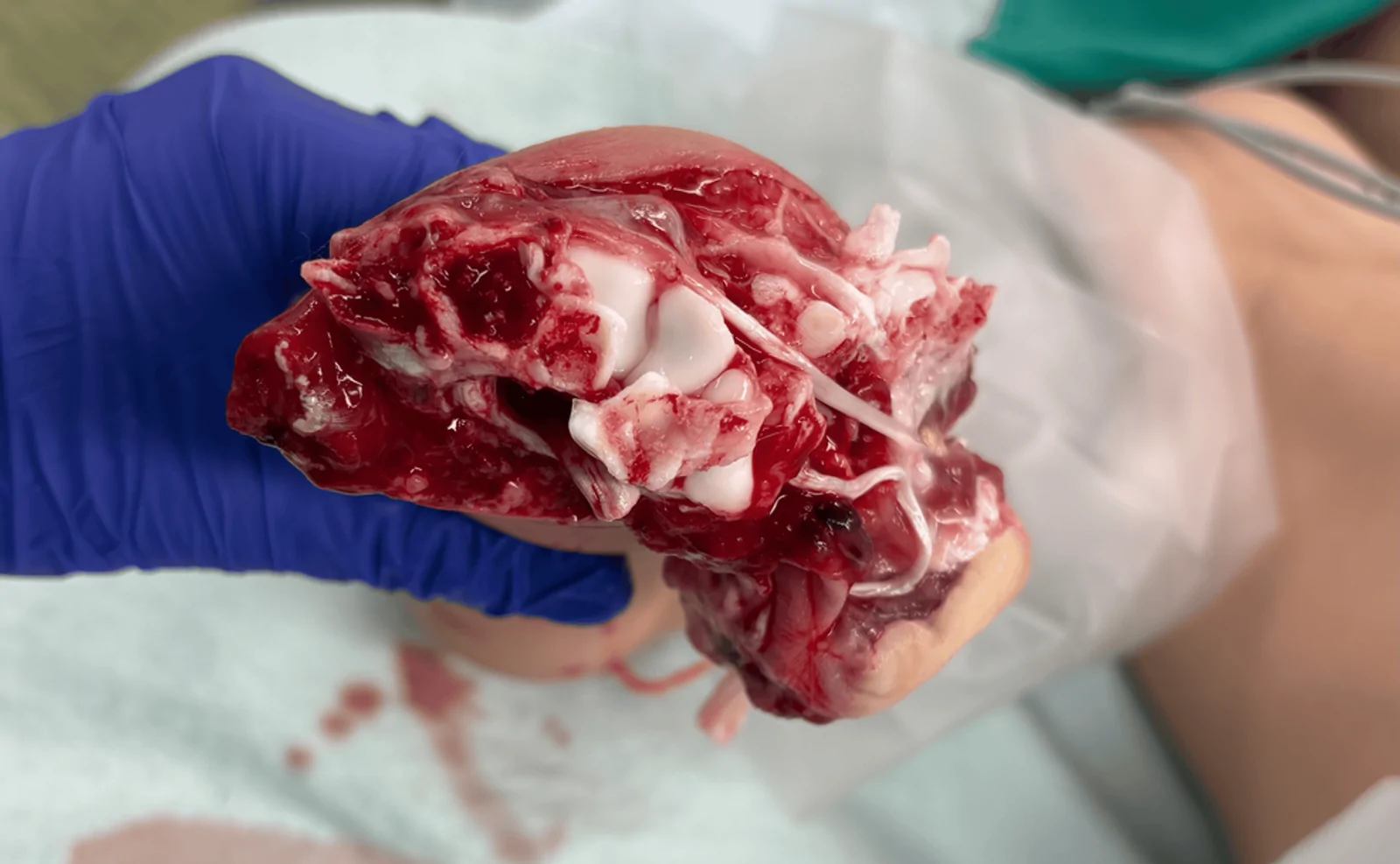
Severely mangled hand to the level of the carpometacarpal joints.

The patient was then immediately returned to the operating room for definitive surgical management. A tourniquet was applied to the upper arm, and the wound was copiously irrigated. Upon inspection, the hand was found to be severely mangled to the level of the carpometacarpal joints, with non-viable soft tissue and comminuted bony injury (Figures 3, 4).

**Figure 5:**
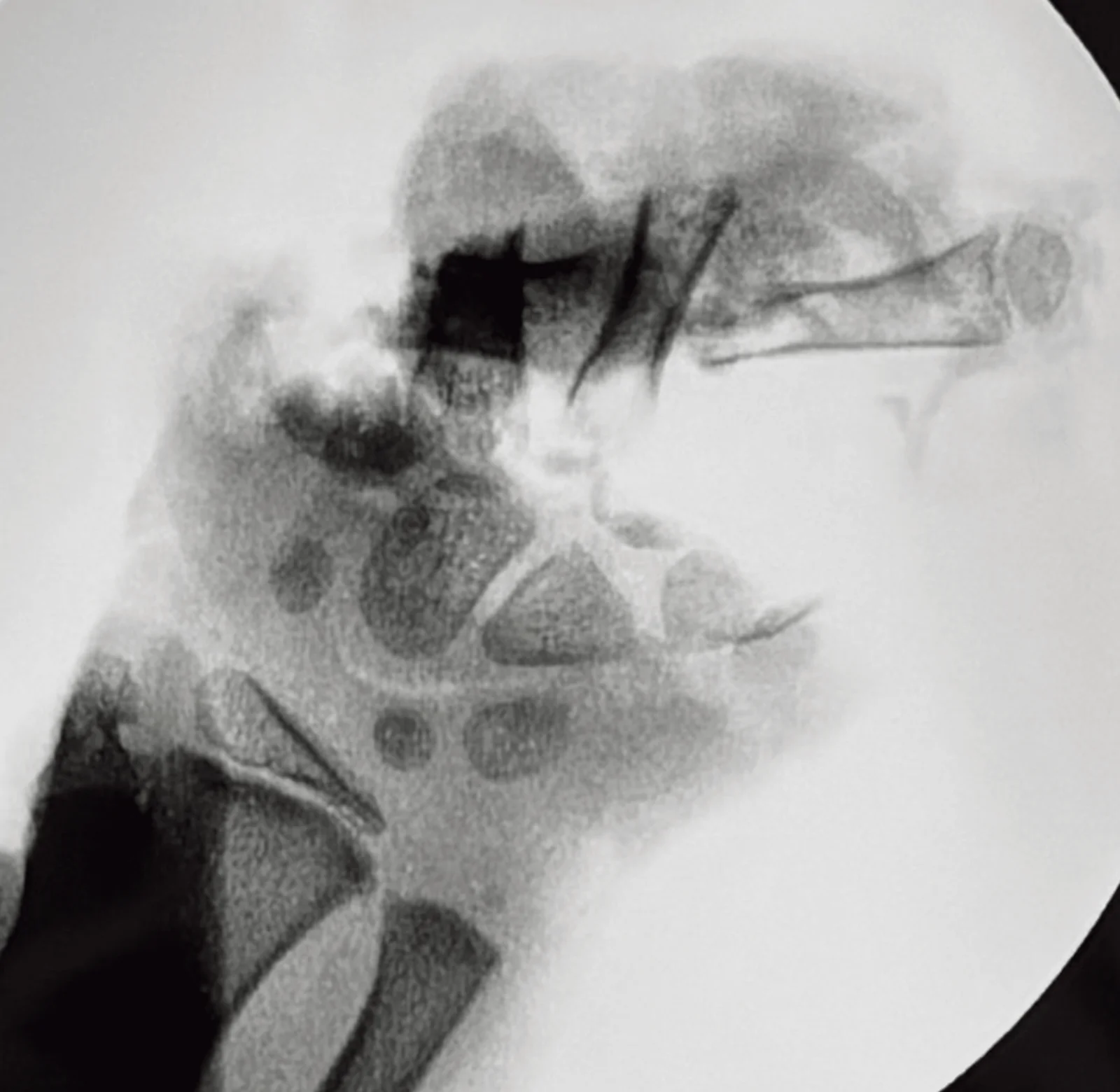
Intraoperative fluoroscopy demonstrating intact carpal bones and a stable distal radioulnar joint, with destruction to the metacarpals and distal structures.

A superficial laceration was also present along the volar wrist. Intraoperative fluoroscopy demonstrated intact carpal bones and a stable distal radioulnar joint, with destruction limited to the metacarpals and distal structures (Figure 5).

**Figure 6:**
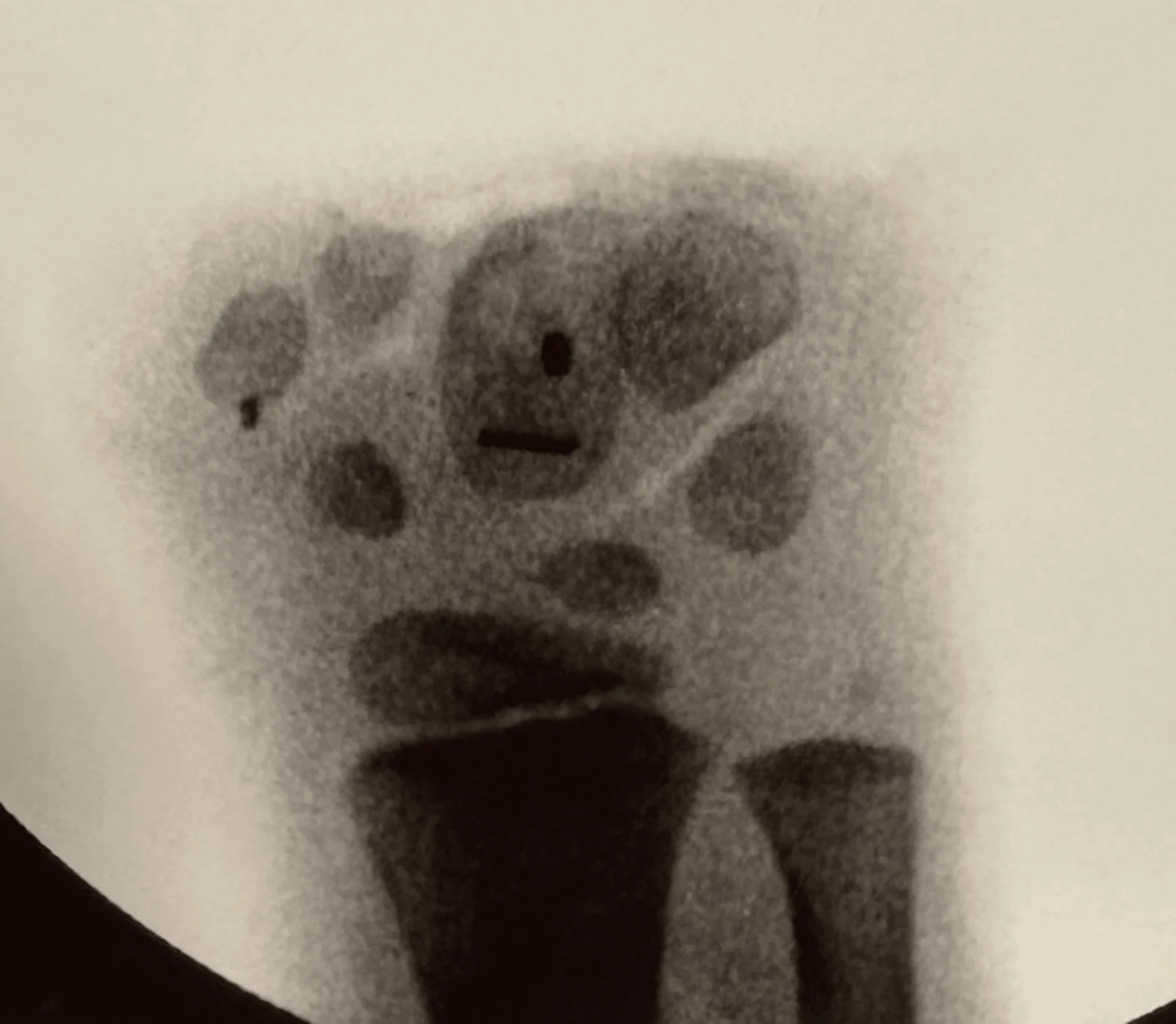
Final fluoroscopic imaging confirming preservation of the carpus and wrist alignment.

Given the extent of tissue loss and non-reconstructable injury, the decision was made to proceed with amputation at the carpometacarpal level. Devitalized bone was excised, and remaining soft tissues were carefully debrided. Final fluoroscopic imaging confirmed preservation of the carpus and wrist alignment (Figure 6).

**Figure 7:**
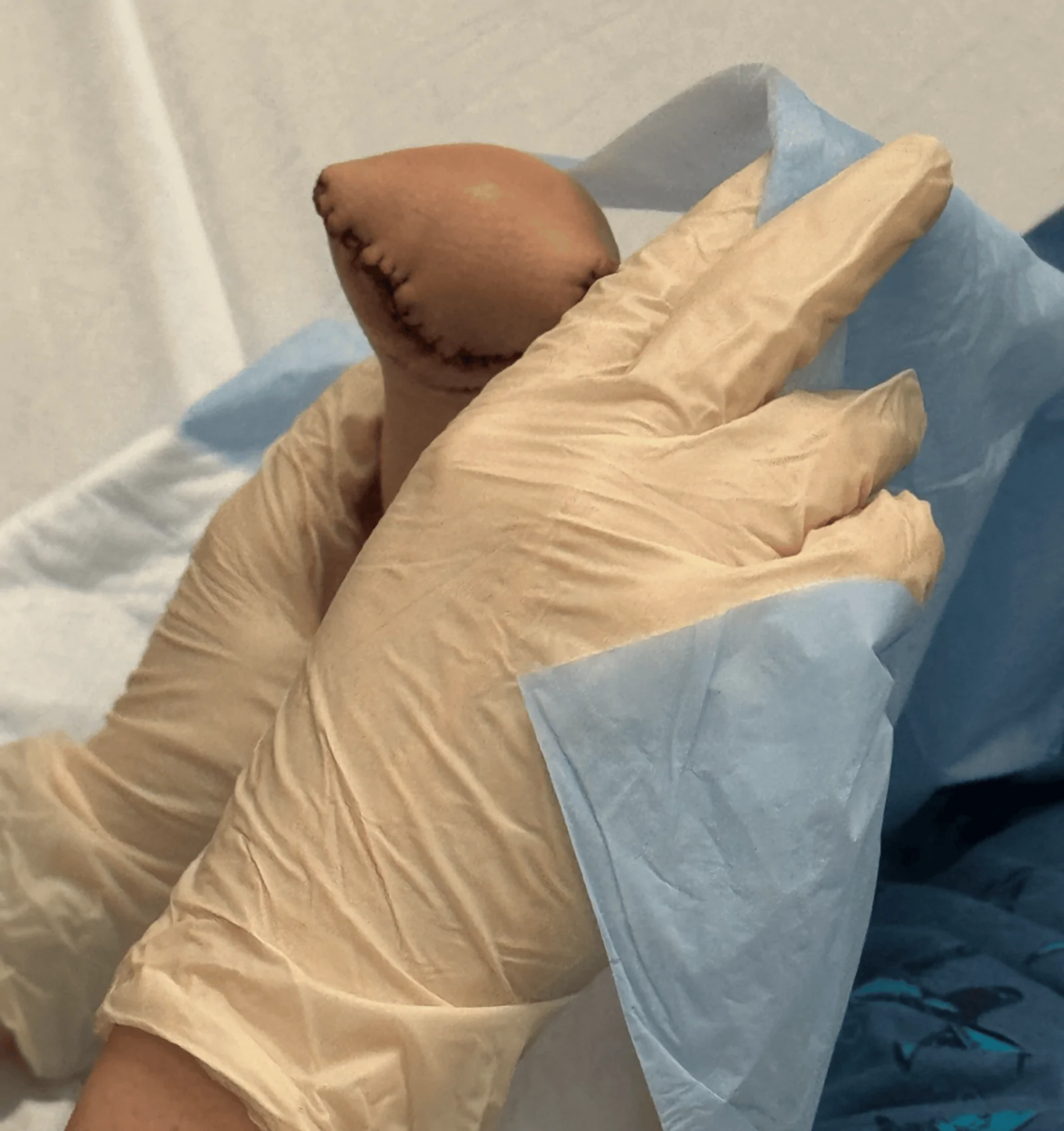
One-week postoperative outcome with viable dorsal skin.

Viable dorsal skin was preserved and advanced to allow primary closure (Figure 7).

The patient tolerated the procedure well and was extubated in the operating room. Estimated blood loss was minimal, and no transfusion was required. A regional anesthetic block was placed for postoperative analgesia, and he was transferred to the postanesthesia care unit in stable condition. He was subsequently admitted for observation and multidisciplinary follow-up including psychology, occupational therapy, rehabilitation, and eventual prosthetic consultation. He was discharged home 72 hours postoperatively and continued close outpatient follow-up with plastic surgery, occupational therapy, social work, psychology, and the physical medicine and rehabilitation team. Early scar massage and desensitization therapy were initiated as part of his rehabilitation. At three months postoperatively, he was successfully fitted with a universal prosthesis, allowing him to resume activities including bicycling and all-terrain vehicle (ATV) riding. He was also fitted with a sport-specific "broomstick" prosthesis to facilitate participation in hockey and lacrosse. Additional adaptive equipment, school accommodations, and clothing modifications were implemented to maximize independence with activities of daily living. At his most recent follow-up, he reports no phantom limb pain or symptomatic neuroma, has adapted well functionally, and is not anticipated to require revision surgery. The timeline of events is displayed in Table 1.

**Table 1:** Detailed timeline of events.

Time	Event
19:00	Household injury
19:20	EMS arrived at scene
19:40	Failed extrication at scene, machine disassembled
20:10	Arrived at hospital, trauma team activation, plastic surgery paged
20:30	Booked and consented for operating room, Level A
21:20	Intubated in operating room
21:40	Failed manual extraction, fire department called
22:00	Fire department arrived, patient moved to nearby room
22:30	Mechanical auger rotation and successful extrication
22:40	Transferred back to operating room and surgery began
0:15	Case completed
72 hours	Discharged home
3 months	Prosthetic use started

## Discussion

Meat grinder injuries represent one of the most destructive mechanisms of upper extremity trauma because they combine crushing, avulsion, torsional, and shearing forces into a single injury pattern. The recent systematic review by Alhusain et al. identified only a small number of published patients over a 50-year period, underscoring the rarity of these injuries despite their severe functional consequences [3]. Most reported patients required extensive debridement or amputation, while microsurgical reconstruction was feasible only in carefully selected cases [1,3,8].

Pediatric patients present unique management challenges. Smaller hands are more readily drawn into household meat grinders, often resulting in injuries extending proximally beyond the digits into the metacarpals or carpus [1,3,9,10]. In addition to the immediate reconstructive challenges, loss of hand function during childhood has lifelong implications for motor development, prosthetic adaptation, education, and psychosocial well-being [10]. Consequently, early involvement of occupational therapists, rehabilitation specialists, psychologists, and prosthetic teams is essential to optimize long-term functional and psychosocial outcomes following definitive treatment.

Our case differs from previously published reports because the principal operative challenge was not reconstructive decision-making, but safe mechanical extrication after conventional methods had failed. Previous reports describe successful release using reverse rotation of the auger mechanism, partial disassembly of the grinder, or cutting of the housing with specialized equipment [1-4,7]. In contrast, the operating room lacked the mechanical tools necessary to safely dismantle the device, requiring intraoperative coordination with municipal emergency services before definitive surgical treatment could proceed. This systems-level challenge has received little attention in the published literature and represents the principal educational value of this report.

Beyond highlighting an uncommon scenario, this case offers several practical lessons for surgeons managing mechanical entrapment injuries. Forceful extraction should be avoided when significant resistance is encountered, as additional traction may exacerbate crush injury and further compromise neurovascular and skeletal structures. Early recognition that specialized rescue equipment or technical expertise may be required should prompt timely involvement of emergency services rather than repeated unsuccessful extraction attempts. Definitive reconstructive decision-making should also be deferred until complete release of the extremity permits accurate assessment of tissue viability and injury extent.

Following successful extrication, intraoperative assessment demonstrated extensive destruction of the metacarpals, non-viable soft tissue, and irreversible injury distal to the carpus. Although microsurgical reconstruction and revascularization have been described in carefully selected patients, successful limb salvage depends on preservation of critical vascular, skeletal, tendon, and soft tissue structures [4,7,8]. In the setting of severe crush-avulsion injury such as that observed in this patient, primary amputation provided the most predictable functional outcome while avoiding multiple unsuccessful reconstructive procedures and prolonged recovery. Although traction neurectomy for visible nerves was performed, additional procedures aimed at neuroma prevention were not performed during the index operation because of the contaminated nature of the injury and the priority of achieving durable soft-tissue coverage. Contemporary techniques such as targeted muscle reinnervation (TMR) or regenerative peripheral nerve interfaces (RPNI) may be considered during delayed stump revision in selected patients to improve long-term pain control and facilitate prosthetic use. Fortunately, the patient has reported no phantom limb pain or symptomatic neuroma during follow-up, although continued surveillance remains appropriate given his young age.

This case also reinforces the importance of multidisciplinary leadership during complex trauma management. Successful treatment depended on effective communication between the surgical, anesthesia, nursing, radiology, and emergency response teams, allowing safe extrication while maintaining patient safety throughout. Although multidisciplinary collaboration was essential, the surgical team remained responsible for coordinating overall management, including recognizing when conventional extraction techniques had failed, engaging external emergency responders, directing definitive operative management following release, and coordinating postoperative rehabilitation. Early involvement of occupational therapy, psychology, and prosthetic services was equally important in facilitating recovery and long-term functional planning. These principles may be broadly applicable to other uncommon mechanical entrapment injuries encountered in tertiary trauma centers.

Finally, this case reinforces the ongoing importance of injury prevention. Although household meat grinders are used less frequently than in previous decades, severe injuries continue to occur in both children and adults [2,3]. Public education regarding supervision of children during food preparation, engineering improvements to grinder safety mechanisms, and continued public awareness campaigns remain important strategies for reducing these preventable injuries [9,11].

## Conclusions

Pediatric hand entrapment within a household meat grinder represents an uncommon but devastating injury requiring prompt multidisciplinary management and thoughtful surgical decision-making. This case demonstrates that successful treatment may depend not only on operative expertise but also on rapid collaboration with external emergency services when conventional operating room resources are insufficient for safe mechanical extrication.

Following successful release of the entrapped extremity, early recognition of non-salvageable tissue permitted timely definitive amputation and facilitated coordinated rehabilitation planning involving occupational therapy, psychology, and prosthetic services. This case highlights important systems-level considerations that may inform future management of rare mechanical entrapment injuries while reinforcing the continued importance of household injury prevention and equipment safety.

## References

[1] Abdelmegeed AG, Saied S, Hifny MA (2023). Meat grinder hand injury in children: a report of 5 cases. Ann Chir Plast Esthet.

[2] Duman İG (2022). Prevalence of household meat grinder-induced severe hand injuries: a retrospective clinical study. Ulus Travma Acil Cerrahi Derg.

[3] Alhusain AM, Alshaalan S, Alkhunein J, Alghamdi A, Alhije F, Alaowid F, Alhujayri AK (2025). Meat grinder-related hand injuries: a 50-year systematic review of case reports and series. Plast Reconstr Surg Glob Open.

[4] Dmytrovych PM, Volodymyrovych HP, Orestovych KI, Georgiyovych DV (2022). Pediatric high-energy and other traumatic injury: cases and reviews. Int J Health Sci.

[5] Lahiri A (2019). Managing mutilating hand injuries. Clin Plast Surg.

[6] Neumeister MW, Brown RE (2003). Mutilating hand injuries: principles and management. Hand Clin.

[7] Ibrahim HS, Ilker E, Taskoparan H (2022). Meat grinder hand injury; case report. Int J Surg Case Rep.

[8] Brandner M, Bunkis J, Trengove-Jones G (1985). Meat grinder injuries to the upper extremity. Ann Plast Surg.

[9] Gebreslassie Kassa B (2017). Meat grinder hand injuries: serial cases. MOJ Clin Med Case Rep.

[10] del Piñal F (2007). Severe mutilating injuries to the hand: guidelines for organizing the chaos. J Plast Reconstr Aesthet Surg.

[11] Patial T, Mittal RK, Garg R, Shah S, Kaur A (2021). Grinder injury of the hand: a rare but devastating occupational hazard. Surg J (N Y).

